# Beyond radiotheranostics: how imaging modality defines theranostics

**DOI:** 10.1186/s41747-026-00823-x

**Published:** 2026-10-08

**Authors:** Irena Pashkunova-Martic, Verena Pichler, Thomas H. Helbich

**Affiliations:** 1 Department of Biomedical Imaging and Image-guided Therapy, Division of Molecular and Structural Preclinical Imaging, Medical University of Vienna & General Hospital of Vienna, Waehringer Guertel 18-20, 1090 Vienna, Austria; 2 Institute of Inorganic Chemistry, Faculty of Chemistry, University of Vienna, Waehringer Strasse 42, 1090 Vienna, Austria; 3 Research Platform Medical Imaging (RPMI), Medical University of Vienna, Waehringer Guertel 18-20, 1090 Vienna, Austria; 4 Department of Pharmaceutical Sciences, University of Vienna, Josef-Holaubek-Platz 2 (UZA II), 1090 Vienna, Austria; 5 Department of Pharmacy, University of Oslo, Problemveien 11, 0313 Oslo, Norway; 6 Joint Applied Medicinal Radiochemistry Facility, University of Vienna and Medical University of Vienna, 1090 Vienna, Austria

**Keywords:** Drug delivery systems, Molecular imaging, Positron emission tomography, Precision medicine, Theranostics

## Abstract

**Abstract:**

Theranostics integrates diagnostic imaging and therapy within a single platform, enabling image-guided treatment delivery, real-time monitoring, and therapeutic optimization. The choice of imaging modality fundamentally shapes the design, functionality, and clinical maturity of theranostic systems. This review examines the theranostic concept through the lens of major imaging modalities employed in clinical and preclinical practice, including positron emission tomography (PET), single-photon emission computed tomography (SPECT), ultrasound (US), optical imaging (OI), magnetic resonance imaging (MRI), and computed tomography (CT). Beyond modality-specific approaches, the review highlights emerging multimodal theranostic strategies that integrate complementary imaging technologies to overcome individual limitations, enhance biological specificity, and improve treatment guidance. For each modality, we discuss the structural components of theranostic agents, their mechanisms of action, and the balance between diagnostic sensitivity and therapeutic efficacy. The review also highlights current and emerging clinical applications across major disease areas, while critically addressing limitations, such as spatial resolution, depth penetration, toxicity, and translational barriers. Overall, this article underscores that theranostics are expanding from modality-defined implementation toward integrated, multimodal strategies that are likely to shape the next generation of personalized and precision imaging-guided therapies.

*****Relevance statement***:**

Imaging modality-driven theranostic design enables more precise, adaptive, and personalized image-guided therapies by aligning diagnostic sensitivity with therapeutic mechanisms, thereby improving treatment selection, response monitoring, and translational integration across clinical disciplines.

**Key Points:**

- Theranostics is determined by the underlying imaging modality rather than by a unified theranostic framework.
- Imaging modality dictates integration strategy.
- Nuclear medicine theranostics are clinically established, while MRI-, CT-, ultrasound-, and optical-based theranostics remain largely translational or function primarily as image-guided therapies.
- Multimodal theranostics address modality-specific limitations.
- Future progress depends on imaging-driven design and standardization.

**Graphical Abstract:**

**Figure:**
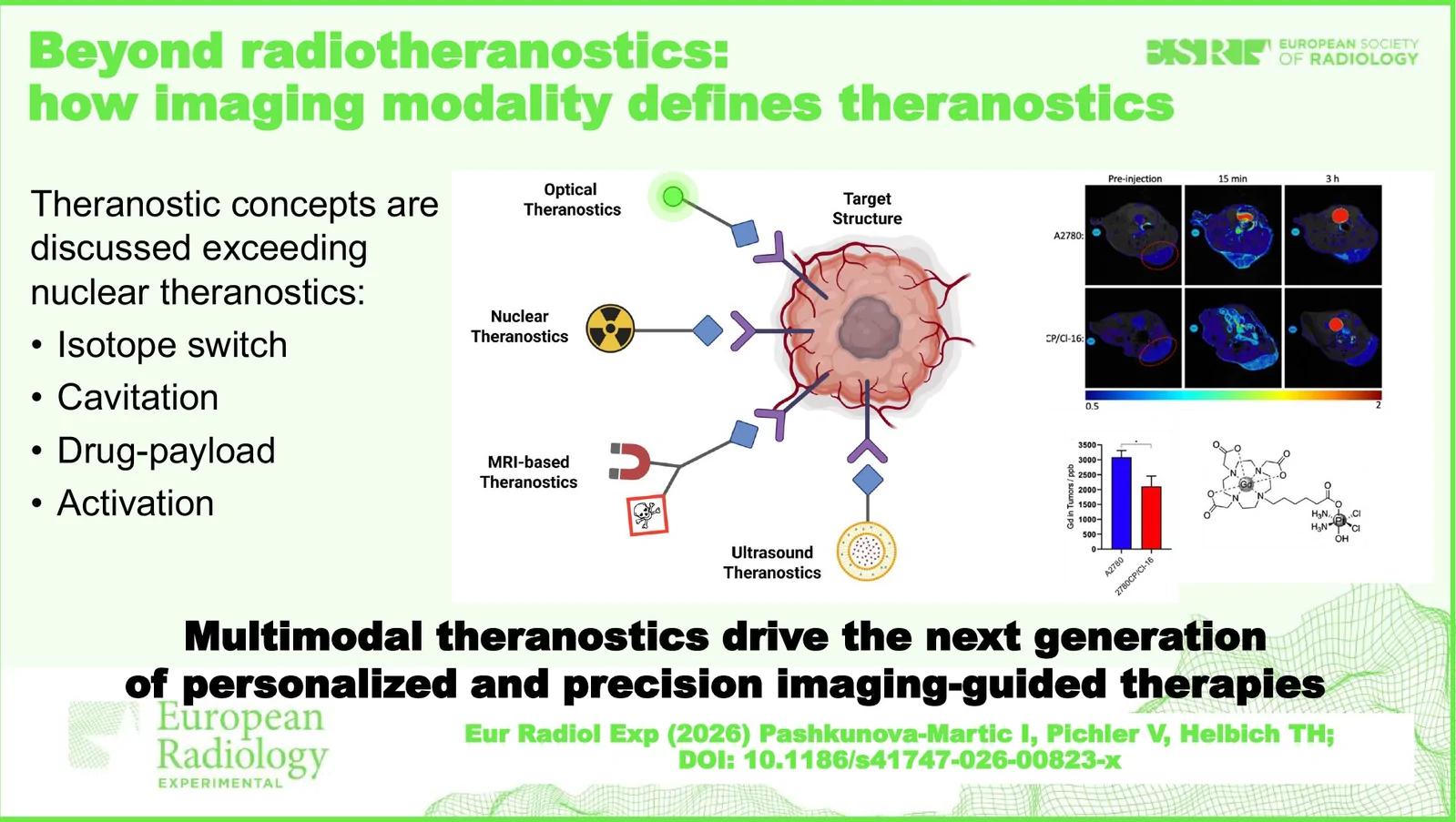

## Background

The theranostic concept has become a major focus in medical imaging, driven, in particular, by the USA Food and Drug Administration approvals of targeted radiopharmaceutical therapies, such as Lutathera [1] and Pluvicto [2], which have demonstrated the clinical potential of combining molecular imaging with personalized treatment. The global theranostics market is projected to expand substantially over the next several years, with revenues expected to grow from approximately USD 2.1 billion in 2023 to around USD 4.3 billion by 2028 at a compound annual growth rate of about 15.5%, reflecting the expanding integration of theranostic approaches across medical imaging and personalized medicine applications [3].

Although the term theranostics was introduced only about thirty years ago, the underlying concept of image-guided therapy has been used in clinical practice for much longer. Radioiodine therapy is one of the pioneering examples [4]. The ^130^I/^131^I treatment scheme was first applied in the early 1940s, with competing groups publishing reports about this therapeutic tool for hyperthyroidism in clinical trials [5, 6], with the first clinical application being published by Seidlin et al [7]. The triumphal march of radioiodine therapy was further developed with the arrival of single-photon emission computed tomography (SPECT) cameras, which allowed the imaging of ^131^I and the monitoring of the therapeutic intervention [8].

The term “theranostics” was first introduced by John Funkhouser in 1998 [9]. It describes a medical strategy that integrates therapeutic interventions and diagnostic tools into a single platform, enabling diagnostic imaging (*e.g.*, tumor detection or inflammation localization), targeted drug delivery, and real-time monitoring of treatment efficacy. Consequently, any diagnostic intervention, whether based on biomarkers (*e.g*., histopathology, liquid biopsies) or image guidance that directly informs therapeutic decision-making, may be considered a theranostic procedure, providing an essential framework for personalized medicine [10].

In this review, we focus on imaging-based therapies that involve chemically vectorized diagnostic and therapeutic entities. However, we wish to draw the reader’s attention to complementary reviews that have addressed related topics, including image-guided interventional procedures [11] or image-guided drug delivery [12]. This success also sparked debate over the appropriate terminology, with some authors proposing theragnostics as a more accurate term [13, 14]. Nevertheless, this alternative did not gain traction, and theranostics remains the term predominantly used in the literature.

## From nuclear medicine to multiple modalities

Originating in nuclear medicine, the concept of imaging-guided theranostics has since expanded across multiple imaging modalities, and this article provides a concise overview of the recent developments [15]. Although routine clinical application remains largely confined to positron emission tomography (PET) and SPECT, radiopharmaceuticals constitute clinically established theranostic platforms with integrated diagnostic and therapeutic platforms, whereas ultrasound and computed tomography (CT) mainly support image-guided interventions and emerging nanoparticle-based approaches. In contrast, magnetic resonance imaging (MRI) remains largely translational despite its potential for targeted and drug-tracking applications, and optical imaging and some multimodal concepts are predominantly preclinical owing to limited tissue penetration.

**Fig. 1:**
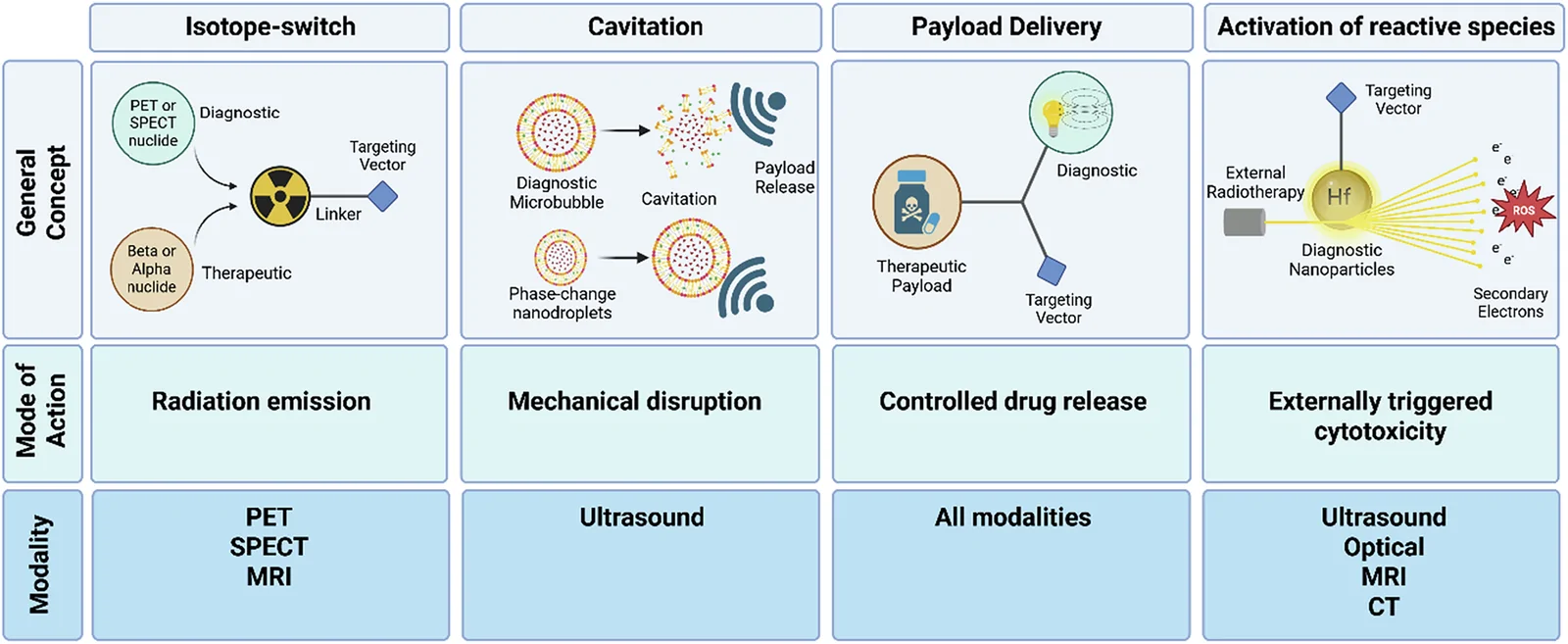
Core theranostic frameworks across imaging modalities. Theranostic systems can be categorized into four principal concepts: isotope-switch strategies in nuclear medicine; cavitation-driven approaches in ultrasound; carrier-based payload delivery systems; and activation-based mechanisms, such as photodynamic or radiosensitization therapies. These frameworks map differently onto imaging modalities, highlighting that theranostics are defined by their underlying mechanism rather than a single unified design principle. Figure created by biorender.com

Overall, theranostic concepts include four main frameworks, including isotope-switch, cavitation, payload delivery, and activation (Fig. 1), with the possibility for multimodality and diverse combinations.

## Nuclear imaging-based theranostics

PET and SPECT-based theranostics can be considered the most advanced and clinically established theranostic approaches, exerting substantial impact and providing inspiration for the broader imaging community. The already mentioned big three theranostics are, without a doubt, radioiodine therapy for thyroid, [^68^Ga/¹⁷⁷Lu]-DOTATATE (Lutathera) for neuroendocrine tumors, and [^68^Ga]PSMA-11/[¹⁷⁷Lu]PSMA-617 (Pluvicto) for metastatic, PSMA-positive prostate cancer. Even if these therapies are the most discussed ones, there are several more that should be mentioned, including [^123^I/^131^I]Iobenguane (MIBG) for neuroendocrine tumors, such as pheochromocytoma, paraganglioma, and certain neuroblastomas [16], as well as radiolabeled antibodies, and ^223^Radium chloride (Xofigo) [17].

The key factor that underlies the success of these theranostic approaches is the versatility of the available radionuclides, the ability to use the same chemical entity as a vector for both diagnosis and therapy, and the minuscule pharmaceutical doses required for application (George de Hevesy’s microdosing and radiotracer concept [18]), as the radiation provides the therapeutic effect, while the vector determines where it acts (Fig. 1). With this, the vector/chemical entity itself does not bear the therapeutic efficacy. Consequently, the choice of which diagnostic radionuclide is paired with which therapeutic one comes down to physico-chemical behavior and reaction chemistry that exhibit pharmacokinetics similar to those of the diagnostic *and* therapeutic radiopharmaceuticals. In the ideal case, such as for the element iodine or copper, the diagnostic and therapeutic isotopes originate from the same element, constituting a so-called “real theranostic pair” (see Table 1), whereas, *e.g.*, the diagnostic radiometal gallium-68 found its partner in lutetium-177, for which the chemical differences are minimal and acceptable. A list of well-described and upcoming pairs can be seen in Table 1 [19].

**Table 1:** Examples of theranostic pairs for PET and SPECT, and possible pairing to a theranostic radionuclide

Diagnostic radionuclides		Therapeutic radionuclides	
Nuclides	Decay mode	Nuclides	Decay mode
^18^F (PET)	β^+^ (97%) EC (3%)	^211^At	α (42%) EC (58%)
^64^Cu (PET)	β^+^ (18%) EC (44%)	^67^Cu	β^-^ (100%)
^68^Ga (PET)	β^+^ (89%) EC (11%)	^177^Lu	β^-^ (100%)
^89^Zr (PET)	β^+^ (23%) EC (77%)	^225^Ac	α (100%)
^99m^Tc (SPECT)	IT (100%)	^90^Y	β^-^ (100%)
^123^I (SPECT)	EC (100%)	^131^I	β^-^ (100%)

The table shows only potential but not exclusive pairings of prominent radionuclides; for further information, see ref. [19]

*EC* Electron capture, *IT* Isomeric transition

Although the targeting vector can be considered a radiation-delivery device, as stated by Hofman [20], the success of theranostic radiotherapy is mainly driven by these vectors. Vectors can be small molecules, peptides, proteins, or antibodies, and they determine the extent of tumor targeting and accumulation at the tumor site. High standardized uptake values (SUVs) of the radiotracer determine whether a radiopharmaceutical becomes a game-changer in nuclear medicine, as described by Hofman’s Hierarchy of SUVs [20]. With the current shift from beta- to alpha-therapy, quantitative imaging and dosimetry prediction further allows the ability to monitor absorbed doses [21]. To find the perfect fit between the vector and the radionuclide, a rule of thumb indicates that the physical (isotope) and the biological half-life (vector) must match. Consequently, small molecules with fast kinetics are often paired with short-lived radionuclides, whereas larger molecules and biologicals require longer-lived radionuclides [22, 23]. Key aspects of radionuclide selection, vector design, and the emergence of targeted alpha therapy have been comprehensively discussed elsewhere [22, 24, 25].

To date, most clinical innovations and applications of radiotheranostics have focused on oncology, but the field is increasingly exploring applications in non-cancerous diseases. One example is radiosynoviorthesis [26], in which radiocolloids are administered intra-articularly to treat inflammatory joints [26]. Other emerging applications, such as in cardiology or neurodegenerative disorders, follow the principle of image-guided therapy: here, SPECT or PET scans inform and guide personalized clinical management, helping to tailor conventional therapeutic interventions [27–29].

### Clinical status

Theranostic radiopharmaceuticals are becoming increasingly established in clinical practice, particularly in oncology, with several radiopharmaceuticals receiving regulatory approval and demonstrating clear benefits in patient management.

### Limitations

Their broader adoption is constrained by high production costs, limited availability, complex logistics, radiation safety requirements, and the need for specialized infrastructure and trained personnel, which can also limit implementation in non-oncologic applications.

## Ultrasound-based theranostics

Ultrasound imaging is one of the most cost-effective imaging modalities and offers a wide range of clinical applications. The observation that gas bodies can scatter ultrasound waves, thereby enhancing contrast and improving imaging quality, led to the development of microbubble-enhanced ultrasound [30]. A major clinical breakthrough was achieved by stabilizing these 1–10 µm microbubbles through encapsulation of gases such as SF₆ or highly fluorinated carbon gases within albumin, lipid, or polymer shells. However, despite their excellent contrast-enhancing properties, fluorinated gases have raised environmental concerns [31].

As a logical next step, microbubbles were functionalized to enable targeted accumulation in regions of pathological interest, thereby enabling molecular ultrasound and opening the door to ultrasound theranostics. Microbubbles have since become a central focus in theranostics, as they can provide both imaging and therapeutic functions. This can occur directly through cavitation and sonoporation effects [32], or by incorporating therapeutic agents into the microbubble shell [33] or core [34].

**Fig. 2:**
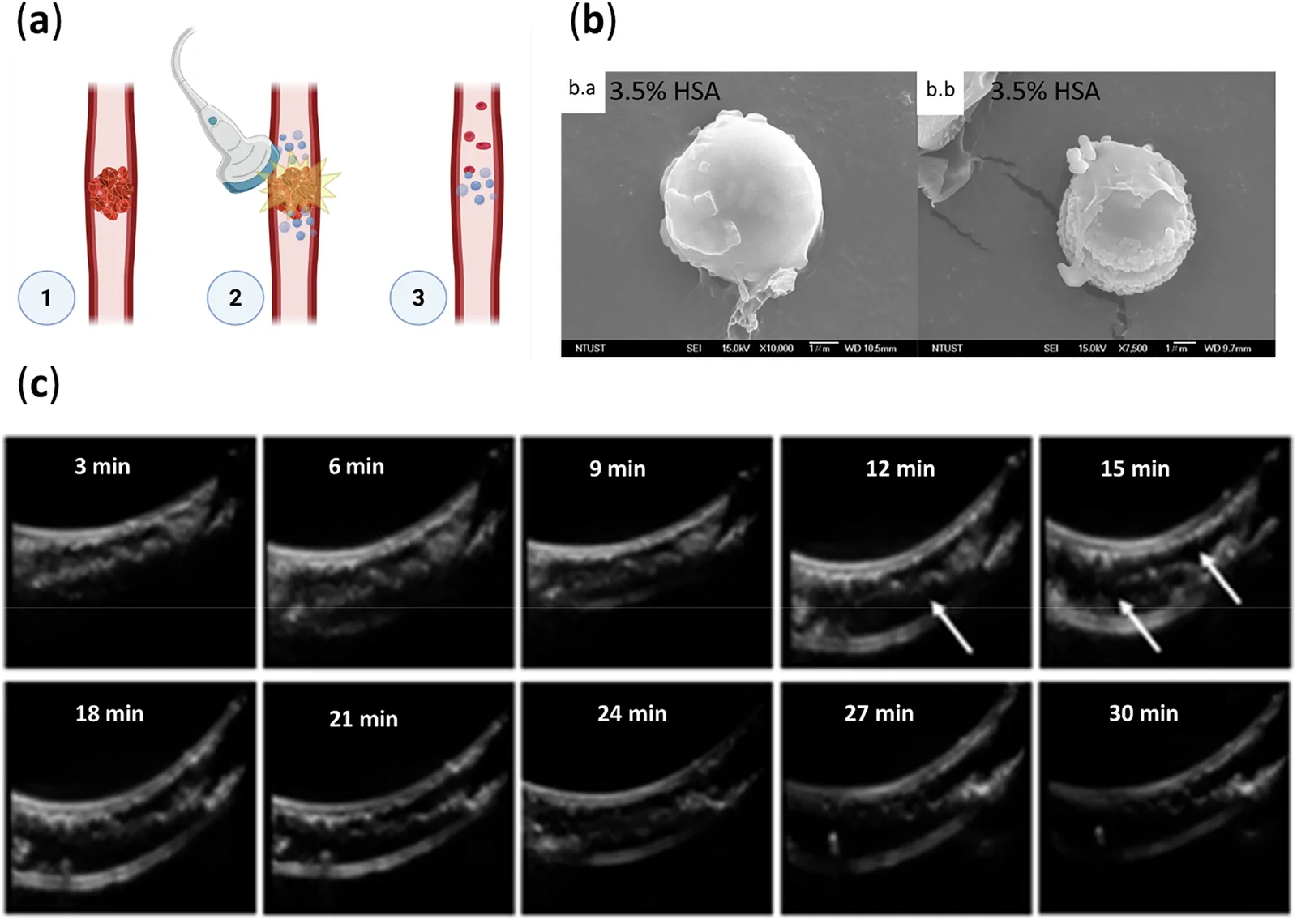
Sonothrombolysis is an example of ultrasound theranostics. **a** The principle of sonothrombolysis includes the application of ultrasound-active microbubbles that are located at the thrombus site (a1). Ultrasound activates cavitation (a2) and the release of the thrombus (a3). Figure created by biorender.com. **b** Scanning electron image of a microbubble. Adapted from ref. [73]; licensed under CC BY-NC 4.0C. **c** Ultrasound images of sonothrombolysis of a blood clot. Adapted from ref. [74]; licensed under CC BY-NC 4.0

Ultrasonic cavitation refers to the formation, growth, and disruptive collapse of vapor-filled bubbles, producing extreme localized effects. The major advantage of microbubbles is that cavitation can be spatially controlled, enabling localized therapeutic action. Once microbubbles reach their target site, incorporated drugs can be released only in the activated area, reducing systemic side effects and enhancing therapeutic efficacy. A notable example of ultrasound theranostics outside of cancer therapy is sonothrombolysis (Fig. 2) [35]. This approach exploits cavitation to mechanically disrupt blood clots, relying solely on the energy released during bubble implosion. As a result, drug loading of the microbubble is not required for therapeutic effect, meaning the same agent serves both diagnostic and therapeutic purposes. Its affordability and effectiveness also make sonothrombolysis suitable for pediatric applications.

The sonoporation effect, on the other hand, refers to the temporary formation of pores in cell membranes, thereby increasing permeability to drug or gene delivery. This effect is primarily a consequence of cavitation rather than an independent mechanism. In the investigational phase, sonoporation is also being explored to open tight junctions in the blood-brain barrier, allowing drugs to reach the brain that would otherwise be unable to cross this critical barrier [36].

More advanced are ultrasound-based theranostic agents developed for cancer therapy and cardiovascular applications, including ultrasound-responsive nanoformulations. While most studies have focused on microbubbles, it is also possible to engineer nanometer-scale ultrasound-responsive agents, such as non-gaseous nanoparticles, nanodroplets, and gas-generating nanoparticles. These particles do not necessarily require a classical core–shell structure; solid nanoparticles can serve as therapeutic carriers, provided they generate a strong physical response to ultrasound. Generally, this requires the particle to have a much higher density and stiffness than water. If the acoustic impedance of the particle closely matches that of water, ultrasound waves pass through without producing sufficient contrast or therapeutic effect [37].

Phase-change nanodroplets are nanoparticles, typically composed of a liquid core, such as a perfluorocarbon, surrounded by a stabilizing shell. Upon exposure to ultrasound, these nanodroplets vaporize into echogenic microbubbles, transitioning from the liquid to the gas phase and expanding from the nanoscale to the microscale [38]. The nanoscale formulation provides several advantages, including enhanced stability, homogeneous suspension, improved tissue distribution, and prolonged biological half-life. In contrast, gas-generating nanoparticles do not increase in size upon activation. Instead, they release ultrasound-detectable gas, such as nitrogen produced through the decomposition of azide groups [39], among other chemical strategies.

A third class of ultrasound-based theranostic agents is used in sonodynamic therapy. In this approach, chemical compounds known as sonosensitizers are applied, most commonly in cancer treatment, to produce cytotoxic effects upon ultrasound activation. Cavitation-induced ultrasonic excitation of these compounds can generate reactive species, including reactive oxygen species or singlet oxygen, which mediate therapeutic effects [40].

### Clinical status

Ultrasound theranostics have achieved substantial clinical translation, largely driven by the approval of microbubble contrast agents for cardiology and liver imaging.

### Limitations

Ultrasound theranostics are constrained by limited penetration depth, acoustic window dependency, and strong operator dependence. Microbubbles are restricted to the intravascular space, and nanoscale agents often show reduced acoustic responsiveness. Cavitation-related off-target effects and challenges in agent complexity, scalability, and regulatory approval further limit translation.

## CT theranostics

The application of CT in the theranostic setting seems underrepresented, despite the fact that CT-based theranostic approaches have successfully advanced to clinical trials [41]. CT-based theranostics are currently implemented primarily through high-Z nanoparticle agents and CT-guided radiotherapy, but CT is best viewed as a structural and quantitative partner in multimodal theranostic systems rather than a standalone platform, similar to optical and MR-based theranostics.

**Fig. 3:**
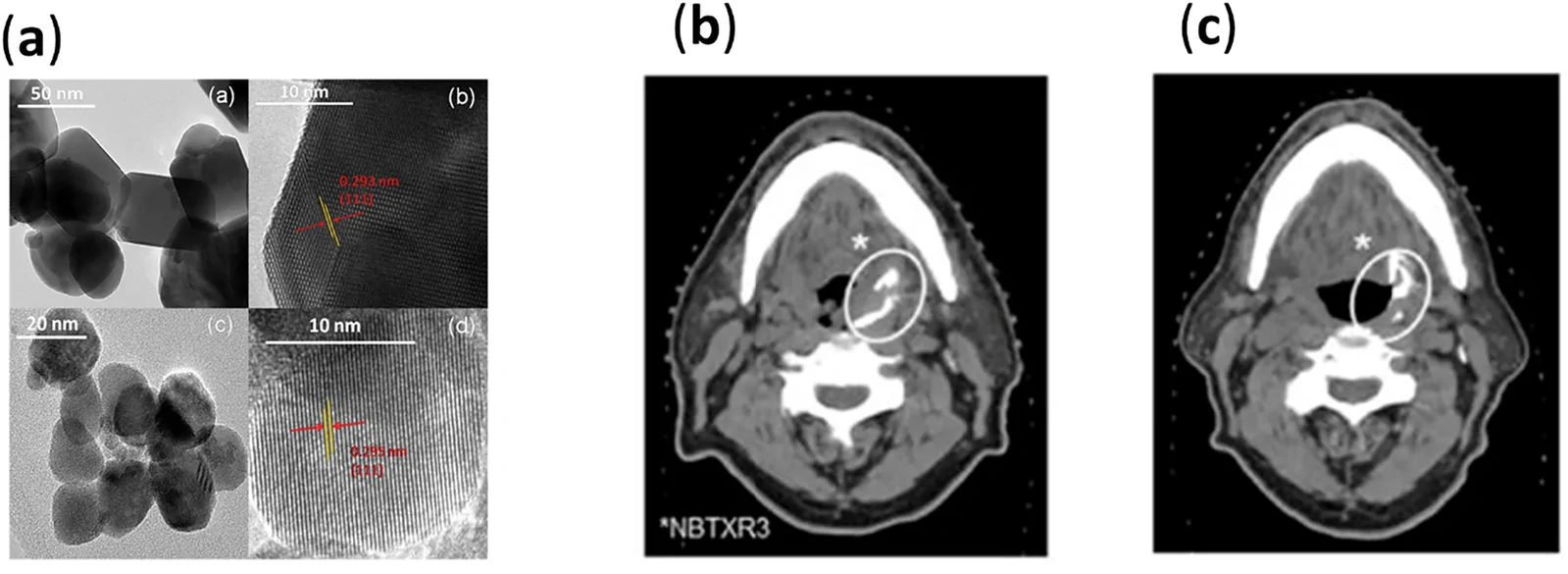
Radiosensitization therapy in clinical trials as an example of CT-based theranostics. **a** Scanning electron microscopy images of HfO_2_-based nanostructures available for radiosensitization in CT imaging. Adapted from reference [75], licensed under CC BY-NC 4.0. CT scan of intratumoral localization of the radiosensitizer platform NBTXR3 after 24 h of injection **b** and after radiosensitization therapy **c**. Adapted from ref. [76], licensed under CC BY-NC 4.0

A potential candidate for a CT contrast agent that can be activated via X-ray attenuation is hafnium oxide (HfO_2_) nanoparticles or a metal-organic framework–MOFs [42]. Hafnium has good physico-chemical properties to allow a highly efficient attenuation of x-rays and, therefore, can function well as a CT contrast agent. In addition, their high atomic number (*Z* = 72), high electron density, and chemically inertness enable them to also be engineered into nanoparticles, including functionalization with targeting vectors. In parallel, Hafnium is also accessible for radiosensitization therapy, as when irradiated (*e.g*., as during external radiotherapy), it produces local dose enhancement via secondary electrons. A recently published clinical trial on NBTXR3-nanoparticles (Fig. 3) in a group cohort of 180 patients validated the mode of action of this radiosensitizer [43].

Another example, although less effective in achieving radiosensitizing effects, is provided by iodinated compounds and nanocarriers or nanoparticles. Honorable mentions are iodine-containing covalent organic frameworks (COFs) that can function as radiosensitizers, circumventing the need for heavy metals [44]. Potassium iodine-coated nanoparticles were also tested *in vivo* for their radioactivation, and the observed DNA damage was attributed to the production of hydroxyl radicals [45].

### Clinical status

Overall, progress toward CT-based theranostics and radiosensitizers has been made; still, examples remain limited. Among these, HfO₂ nanoparticles appear particularly promising, as they have already advanced to clinical trials.

### Limitations

The CT technique has relatively low molecular sensitivity, necessitating the use of high concentrations of heavy-metal-based materials. This raises concerns regarding systemic exposure, long-term retention, and dose-related toxicity, particularly for nanoparticle-based agents. Therapeutic efficacy is largely confined to radiosensitization during external beam irradiation, limiting standalone therapeutic capability and temporal control.

## Magnetic resonance-based theranostics

**Fig. 4:**
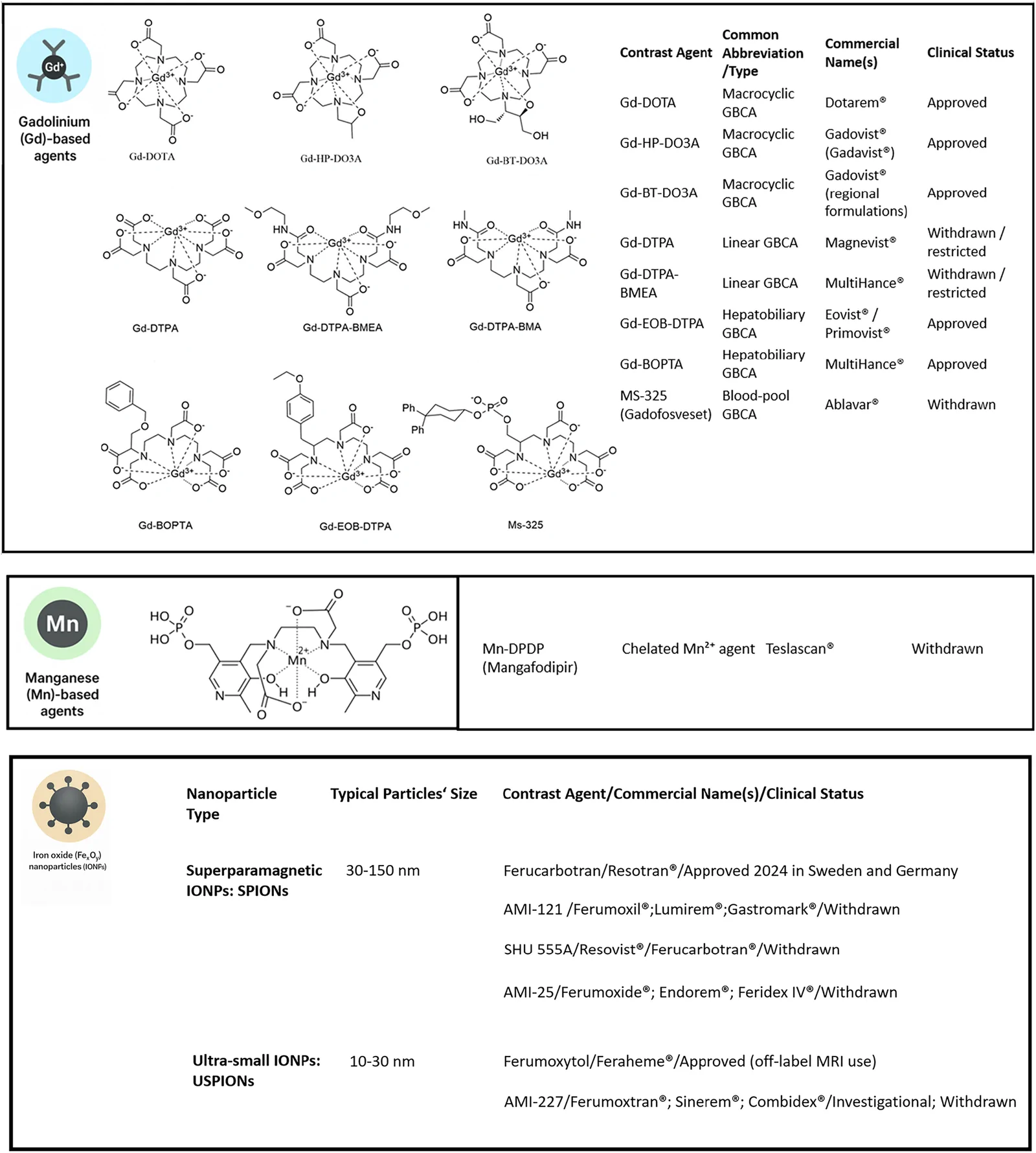
Representative chemical structures, type, commercial names, and clinical status of approved MRI contrast agents, illustrating the three major classes: gadolinium (Gd³⁺)-based chelates for T₁-weighted imaging; manganese (Mn²⁺)-based complexes for T₁-weighted imaging with potential biological activity; and iron oxide–based nanoparticles for T₂/T₂*-weighted contrast. These agents constitute the structural foundation of current MRI theranostic platforms. Adapted with permission from Xiao et al ref. [77] and Karlsson et al ref. [47]

In theranostic applications, gadolinium (Gd) chelates are frequently incorporated into nanocarriers or conjugated to targeting ligands to enable combined imaging and therapy. Manganese (Mn²⁺)-based agents provide an alternative to Gd³⁺, particularly in patients with impaired renal function, and enable responsive or activatable imaging strategies due to their participation in biological redox processes. Iron oxide nanoparticles, including superparamagnetic iron oxide nanoparticles–SPIONs, predominantly act as T₂/T₂* contrast agents and additionally serve as versatile theranostic platforms for drug delivery, magnetic targeting, and hyperthermia. Figure 4 gives an overview of clinically approved MRI contrast agents used as part of the theranostic platforms. Notably, mangafodipir–MnDPDP, the only manganese-based MRI contrast agent approved for clinical use, was withdrawn for commercial reasons but later shown to exhibit antioxidant and superoxide dismutase–like activity, prompting investigation of therapeutic applications, such as cardioprotection, during percutaneous coronary intervention [46, 47].

Effective integration requires balancing imaging sensitivity, pharmacokinetics, targeting specificity, therapeutic efficacy, and safety to support clinical translatability. Core integration strategies include covalent conjugation of therapeutic payloads to MRI contrast agents or carrier scaffolds, encapsulation of contrast agents and payloads within MRI-visible nanocarriers, and coordination-based constructs in which therapeutic payloads act as ligands for paramagnetic metal centers [48–50]. In addition, responsive or activatable systems allow imaging contrast or payload activity to be triggered by disease-associated stimuli, such as acidic pH, oxidative stress, hypoxia, or enzymatic activity, thereby enhancing specificity and reducing off-target effects [51].

MRI theranostic systems have, so far, incorporated a broad range of therapeutic payloads into MRI-visible carriers. Established payloads include chemotherapeutic agents, genetic vectors, photothermal or photodynamic compounds, radiosensitizers, immunomodulators, anti-angiogenic agents, and hyperthermia-inducing materials. The field of MRI theranostics can be broadly divided into (i) molecular MRI theranostics and (ii) MRI nanotheranostics because these two groups differ fundamentally in their scale, mechanism of action, imaging readouts, and translational pathways, despite sharing the overarching goal of integrating diagnosis and therapy.

Compared with nuclear or optical approaches, MRI theranostics offer high spatial resolution, deep tissue penetration, and the possibility of repeated longitudinal assessment without ionizing radiation.

**Fig. 5:**
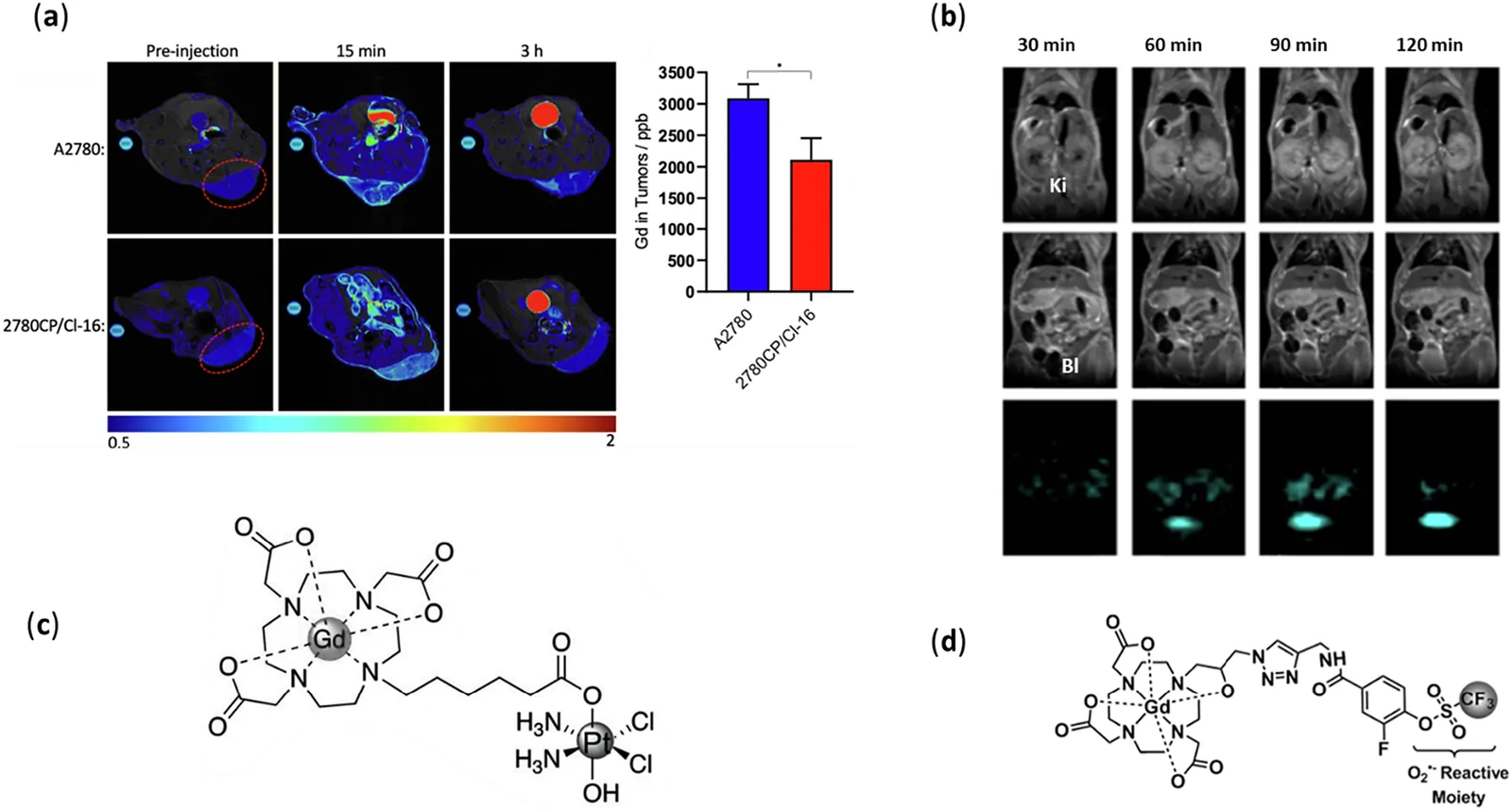
Representative *in vivo* molecular MRI theranostics probes, including (**a**, **c**) a Gd(III)–Pt(IV) conjugate exhibiting cisplatin-like cytotoxicity with T1-weighted MR contrast enhancement in mice bearing cisplatin-resistant 2780CP/Cl-16 and cisplatin-sensitive A2780 tumors. **b**, **d** An activatable molecular ^19^F MRI probe demonstrated *in vivo* by corresponding ^1^H MRI (first and second rows) and ^19^F MRI (third row) acquired at the indicated time points following intravenous administration. Signal enhancement demonstrated in the kidneys (Ki) and in the bladder (Bl) of treated mice. Reproduced with permission from refs. [78, 79]

Molecular MRI theranostics are based on small-molecule or coordination-based agents in which the imaging and therapeutic functions are intrinsically linked at the molecular level (Fig. 5). These systems typically rely on paramagnetic ions (*e.g*., Gd³⁺, Mn²⁺) or nuclei, such as ¹⁹F, either as part of chelated complexes or molecular assemblies, and often resemble conventional MRI contrast agents in size, pharmacokinetics, and biodistribution.

From a clinical perspective, molecular MRI theranostics are conceptually closely aligned with contrast-agent-based diagnostics, offering predictable pharmacokinetics, deep tissue penetration, and the possibility of repeated longitudinal imaging without ionizing radiation. These properties support controlled, mechanism-driven applications and facilitate integration into established imaging workflows.

In contrast, MRI nanotheranostics are based on nanoscale carrier systems, such as polymeric nanoparticles (*e.g.*, poly(lactic-co-glycolic acid)–PLGA) nanoparticles [52], inorganic nanoparticles (*e.g.*, iron oxide nanoparticles) [53], hybrid nanostructures, or dendrimer-based constructs [54] that physically integrate MRI contrast components with therapeutic payloads. In these platforms, imaging and therapy are functionally integrated but not necessarily chemically identical, providing flexibility in payload selection, release kinetics, and multifunctionality [53].

Magnetite (Fe₃O₄) nanoparticles are well-established T₂/T₂* contrast agents and are among the most advanced classes of MRI nanotheranostics. The USA Food and Drug Administration-approved iron oxide formulation, ferumoxytol, has been repurposed for vascular and inflammatory imaging, while preclinical studies exploit magnetite nanoparticles for MRI-guided hyperthermia and drug delivery [55, 56]. Beyond diagnostics, iron oxide nanoparticles have enabled theranostic hyperthermia applications, exemplified by NanoTherm^®^, approved by the EMA for brain tumors and under evaluation for prostate cancer ablation (NCT05010759), and Magnablate^®^ (NCT02033447).

Gadolinium oxide (Gd₂O₃) nanoparticles exhibit high T₁ relaxivity due to their elevated gadolinium content and have been investigated preclinically for MRI-guided cancer therapy, including drug delivery and radiosensitization. Although clinical translation has not yet been achieved, these systems demonstrate strong contrast enhancement at reduced gadolinium doses, underscoring their theranostic potential [57].

Additional nanotheranostic strategies include pH-responsive nanoparticles that enable functional MRI of acidic tumor microenvironments while triggering therapy through increased relaxivity or selective ion release, as well as hybrid gold/iron oxide (Au/FeO) nanoparticles that combine MRI contrast with photothermal therapy for image-guided tumor ablation [58]. Finally, poly(amidoamine)–PAMAM dendrimer-coated iron oxide nanoparticles offer highly tunable surfaces for drug and gene delivery, enabling efficient cellular uptake and MRI-based biodistribution tracking in preclinical studies, although toxicity and scalability remain key challenges [59].

### Clinical status

Clinical implementation remains limited, largely restricted to systems derived from established contrast agents, particularly iron oxide–based formulations such as ferumoxytol.

### Limitations

The comparatively low sensitivity of MRI requires high local concentrations of contrast agents, which limits the feasibility of molecular theranostics with therapeutically relevant payloads and favors nanoscale systems with their associated complexity. Despite persistent challenges, advances in chemistry, bio-responsive materials, and AI-driven quantitative MRI are rapidly redefining the translational landscape of MRI theranostics. As a result, MRI is poised to evolve into a cornerstone of personalized, image-guided therapy, enabling adaptive, patient-specific treatment strategies.

## Optical theranostics

Optical imaging operates on principles similar to those of ultrasound imaging. However, ultrasound remains more clinically established due to its lower cost, greater accessibility, and broad applicability across a wide range of medical settings. Optical imaging in contrast, offers excellent molecular sensitivity and a unique capacity for activatable and mechanism-driven theranostics. However, it is inherently constrained by limited penetration depth due to light scattering and absorption [60]. As a result, optical theranostics are predominantly applied in superficial tissues, endoscopic settings, and intraoperative environments, or are incorporated as complementary components within multimodal theranostic systems, so-called light-mediated minimally invasive theranostics [61].

**Fig. 6:**
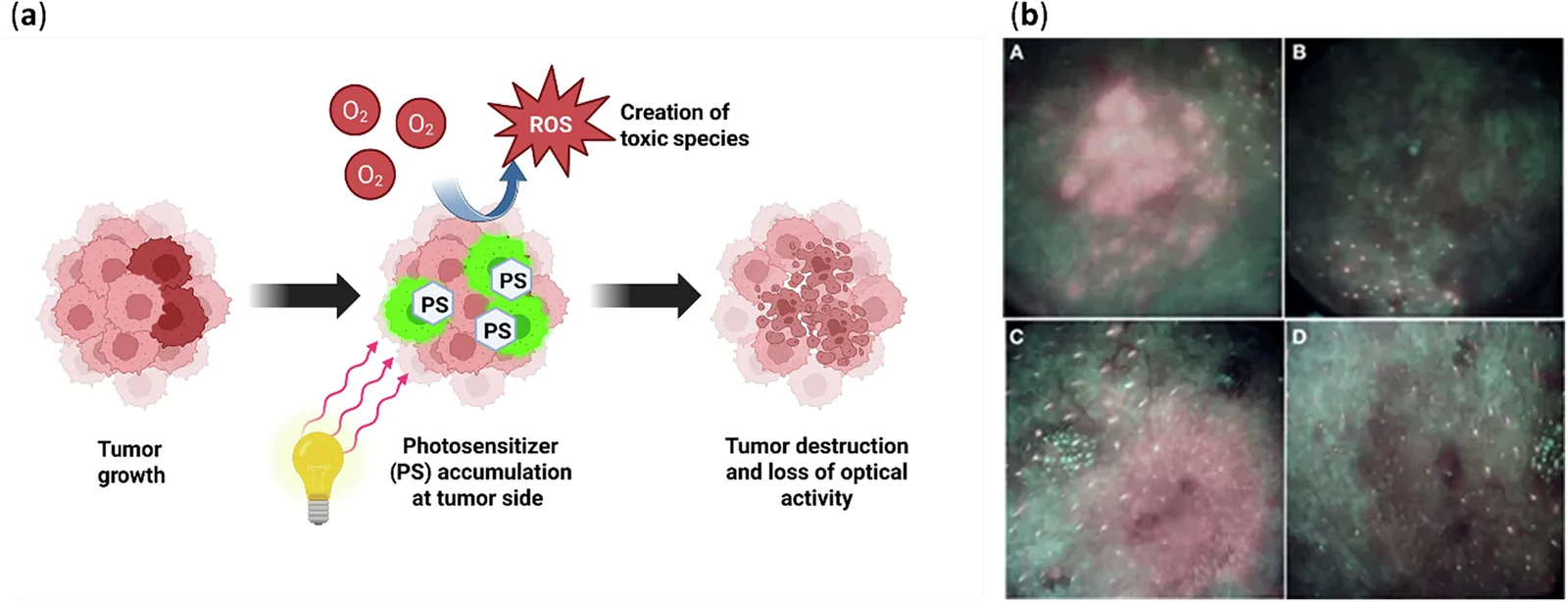
An example of an optical imaging-based theranostic. **a** Concept of a photoactivation therapy. Figure created by biorender.com. **b** Fluorescence images of a basal cell carcinoma before and after illumination. Adapted from ref. [64], licensed under CC BY 4.0

Fluorescent drug conjugates comprise a molecular optical theranostic strategy in which therapeutic agents are covalently linked to fluorescent dyes, enabling direct visualization of drug delivery, biodistribution, and target engagement. Near-infrared fluorophores are commonly employed to reduce tissue autofluorescence and improve penetration depth. In some cases, the fluorophore contributes directly to therapeutic activity, for example, through phototoxic effects or by enabling light-triggered activation. These activatable or “smart” optical probes are designed to generate a fluorescent signal only in response to disease-associated stimuli, such as enzymatic activity, acidic pH, hypoxia, or redox changes [62]. This photodynamic therapy represents a prototypical optical theranostic modality in which the same photosensitizer mediates both imaging and therapy. Upon excitation with light of an appropriate wavelength, photosensitizers generate, *e.g*., reactive oxygen species that induce localized cytotoxicity. Many clinically used photosensitizers exhibit intrinsic fluorescence, enabling visualization of agent accumulation and spatially confined activation [63]. Photodynamic therapy (Fig. 6) has achieved clinical adoption in dermatology, ophthalmology, and oncology, particularly for superficial or endoluminal lesions [64].

The theranostic character of optical imaging arises from the direct coupling of optical imaging activation and therapy. In theranostic designs, probe activation may simultaneously trigger therapeutic payload release or cytotoxicity, enhancing specificity and minimizing off-target effects. These systems enable functional imaging of pathological microenvironments while initiating therapy. However, sensitivity to physiological variability, limited penetration depth, and challenges in robust clinical validation have still confined optical theranostics to predominantly preclinical research. The concept can also be applied to photothermal theranostics, which rely on agents that convert absorbed light into heat to induce localized thermal ablation. Typical photothermal agents include gold nanoparticles, carbon-based nanomaterials, and organic dyes with strong NIR absorption [65]. Many of these agents provide intrinsic optical contrast, enabling image-guided treatment planning and monitoring. While photothermal therapy offers precise spatial control, its efficacy depends on efficient light delivery and sufficient local agent accumulation, resembling CT-based radiosensitizers that rely on external energy input.

Other nanoparticle-based optical theranostics integrate fluorescent imaging agents and therapeutic payloads within nanoscale carrier systems, such as polymeric nanoparticles, liposomes, dendrimers, inorganic nanoparticles, or hybrid constructs [66], comparable to the payloads of ultrasound theranostics. These platforms provide high payload capacity, protection of labile therapeutics, and tunable surface functionalization with targeting ligands. Optical contrast may arise from encapsulated dyes or intrinsically fluorescent nanomaterials, while therapeutic functions include chemotherapy, gene delivery, immunomodulation, photodynamic therapy, or photothermal therapy. Imaging and therapy are functionally integrated but not stringently chemically identical, enabling flexibility at the cost of increased complexity. Pharmacokinetics, biodistribution, long-term safety, and scalability remain major translational challenges, and optical nanotheranostics are frequently combined with MRI or CT to compensate for limited penetration depth [36].

### Clinical status

Clinically, optical theranostics have achieved selective adoption. Photodynamic therapy is an established application supported by approved dyes and photosensitizers. In contrast, most nanoparticle-based, activatable, and multifunctional optical theranostic systems remain at the preclinical or early translational stage.

### Limitations

Optical theranostics are limited by their tissue penetration, which restricts applications to superficial tissues, intraoperative guidance, or endoscopic procedures. Additional challenges include photobleaching, limited quantitative accuracy, potential phototoxicity, and the complexity of designing multifunctional probes. Optical imaging is best regarded as a complementary theranostic modality, similar to CT, rather than a standalone platform, with its strengths most effectively leveraged in multimodal theranostic systems.

## Multimodal theranostics

Multimodal theranostics aim to integrate complementary imaging modalities and therapeutic functions within a single platform to overcome the intrinsic limitations of individual techniques (Table 2). In this context, photoacoustic imaging and Raman-based approaches, including surface-enhanced Raman scattering (SERS), represent emerging multimodal theranostic strategies that combine optical contrast with alternative signal transduction mechanisms, thereby extending the capabilities of established modalities, such as MRI, CT, and PET.

**Table 2:** Comparison of the theranostic concepts across major imaging technologies

Feature	PET/SPECT [70]	Ultrasound [71]	MRI [72]	Optical imaging [66]	Computed tomography [43]
Molecular sensitivity	Very high (pM–nM)	Low–moderate	Low (µM–mM)	Very high (pM–nM)	Very low (mM)
Whole-body imaging	Yes	Limited	Yes	No	Yes
Quantification	Excellent, absolute	Limited/semi-quantitative	Moderate–good	Limited/relative	Excellent (HU-based)
Similarity of diagnostic *versus* therapeutic agents	High (same vector, different radionuclide)	Moderate (drug-loaded microbubbles)	Low–moderate	Moderate	Low
Clinical status	Established clinical routine	Clinical guidance, limited theranostics	Limited clinical translation	Mostly preclinical	Early clinical trials (radiosensitizers)
Limitations	Radiation exposure, cost	Depth, operator dependence	Low sensitivity, safety	Limited penetration	Low sensitivity, high dose

*MRI* Magnetic resonance imaging, *PET* Positron emission tomography, *SPECT* Single-photon emission computed tomography

Photoacoustic theranostics exploit the conversion of absorbed pulsed optical energy into acoustic waves via thermoelastic expansion, effectively combining the high molecular sensitivity of optical absorption with the penetration depth and spatial resolution of ultrasound. Compared with MRI and CT, photoacoustic imaging offers higher sensitivity to endogenous and exogenous chromophores without ionizing radiation, while providing higher spatial resolution than PET at shallow to intermediate depths (Table 2). In theranostic applications, photoacoustic contrast agents frequently overlap with photothermal or photodynamic therapeutic agents, including organic dyes, gold nanoparticles, carbon-based nanomaterials, and semiconducting polymers [66, 67]. These agents enable image-guided photothermal or photodynamic therapy, with photoacoustic imaging providing real-time feedback on agent accumulation, tissue oxygenation, and treatment response. While photoacoustic theranostics partially overcome the depth limitations of purely optical imaging, penetration remains inferior to MRI, CT, and PET, and current clinical applications are largely restricted to superficial tissues, breast imaging, and interventional guidance [66].

Raman- and SERS-based theranostics provide exceptionally high molecular specificity based on vibrational “fingerprint” spectra. Unlike MRI, CT, or PET, which rely on bulk physical or radioactive properties (Table 2), Raman/SERS techniques enable highly multiplexed molecular detection with minimal spectral overlap and excellent chemical discrimination [68]. SERS-based theranostic platforms typically employ plasmonic nanoparticles, most commonly gold or silver, functionalized with targeting ligands and therapeutic payloads [68, 69]. These systems can support image-guided photothermal therapy, drug delivery, and intraoperative tumor margin detection with unmatched specificity. In contrast to PET, Raman/SERS approaches do not require radionuclides but lack whole-body imaging capability and the quantitative depth penetration of nuclear imaging modalities (Table 2).

### Clinical status

Currently, their application is limited to basic research questions.

### Limitations

Penetration depth remains limited compared to MRI, CT, and PET, confining clinical applications to surface-accessible, endoscopic, or intraoperative settings. Photoacoustic and Raman/SERS theranostics are best positioned as complementary components within multimodal platforms, where they add functional or molecular specificity to structurally and quantitatively robust modalities, such as MRI, CT, and PET.

## Conclusions and outlook

Overall, theranostic concepts across imaging modalities are largely based on four core frameworks: the isotope-switch principle established in nuclear medicine; cavitation-mediated mechanisms in ultrasound; carrier-based systems for targeted therapeutic payload delivery; and activation-driven strategies (*e.g*., photodynamic, photothermal, or radiosensitization approaches) that generate reactive species—either applied individually or combined within multimodal platforms. The success of the theranostic concept across imaging modalities is undeniable and continues to expand beyond its nuclear medicine origins. While nanoparticle-based, optical, MRI-, and CT-guided systems present scientific and translational challenges, these areas represent active frontiers of innovation rather than limitations. Rapid advances in vector engineering, smart and scalable material platforms, quantitative imaging technologies, and regulatory harmonization are steadily accelerating clinical translation.

As these developments mature, multimodal theranostics are increasingly positioned to play a central role in precision medicine, enabling more personalized, adaptive, and effective therapeutic strategies.

## Data Availability

Not applicable. This article is a narrative review and does not report original datasets.

## Abbreviations

CT: Computed tomography
HfO_2_: Hafnium oxide
MRI: Magnetic resonance imaging
PET: Positron emission tomography
SERS: Surface-enhanced Raman scattering
SPECT: Single-photon emission computed tomography
SUV: Standardized uptake values
